# Relationship between donepezil and fracture risk in patients with dementia with Lewy bodies

**DOI:** 10.1111/ggi.14929

**Published:** 2024-06-26

**Authors:** Shoya Matsumoto, Mitsutaka Yakabe, Tatsuya Hosoi, Kenji Fujimori, Junko Tamaki, Shunichi Nakatoh, Shigeyuki Ishii, Nobukazu Okimoto, Masahiro Akishita, Masayuki Iki, Sumito Ogawa

**Affiliations:** ^1^ Department of Geriatric Medicine, Graduate School of Medicine The University of Tokyo Tokyo Japan; ^2^ Tohoku University School of Medicine Department of Health Administration and Policy Sendai Japan; ^3^ National Database Japan – Osteoporosis Management (NDBJ‐OS) Study Group Kindai University Faculty of Medicine Osaka Japan; ^4^ Department of Hygiene and Public Health, Faculty of Medicine Osaka Medical and Pharmaceutical University Osaka Japan; ^5^ Department of Orthopedic Surgery Asahi General Hospital Toyama Japan; ^6^ Department of Regulatory Science, School of Pharmacy Tokyo University of Pharmacy and Life Sciences Tokyo Japan; ^7^ Okimoto Clinic Hiroshima Japan; ^8^ Kindai University Faculty of Medicine Osaka Japan

**Keywords:** dementia with Lewy bodies, donepezil, fracture

## Abstract

**Aim:**

Patients with dementia with Lewy bodies (DLB) are at a high risk for falls and fractures. Although cholinesterase inhibitors reportedly are effective in suppressing the progression of cognitive symptoms in DLB patients, their effects on fracture risk remain unclarified. This study aimed to evaluate the association between donepezil use and hip fracture risk in older patients with DLB.

**Methods:**

Using the Japanese insurance claim database, we collected the data of patients aged ≥65 years with DLB from April 2012 to March 2019. After propensity score matching, we compared the fracture rate over 3 years between DLB patients receiving donepezil and those not receiving antidementia drugs.

**Results:**

Altogether, 24 022 239 individuals aged ≥65 years were newly registered from April 2012 to March 2016 and had verifiable information from 6 months before to 3 years after the registration. We identified 6634 pure‐DLB patients and analyzed the data of 1182 propensity score‐matched pairs. The characteristics, including age, sex, fracture history, osteoporosis, and bone mineral density test rate, of the two groups were well balanced by propensity score matching. The incidence rate of hip fracture was significantly lower in DLB patients receiving donepezil than in those not receiving antidementia drugs (0.60 vs. 1.44/100 person‐years, *P* < 0.001), whereas that of vertebral fractures was the same.

**Conclusions:**

Donepezil administration in Japanese people aged ≥65 years with DLB was significantly associated with a decreased risk of hip fracture. Donepezil may provide new benefits to DLB patients. **Geriatr Gerontol Int 2024; 24: 782–788**.

## Graphical abstract

This retrospective cohort study, using a nationwide health insurance claims database, revealed an association between donepezil use and decreased risk of hip fracture in patients aged ≥65 years who had dementia with Lewy bodies.

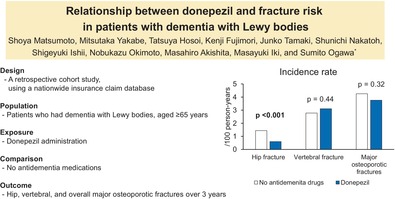

## Introduction

Dementia with Lewy bodies (DLB) is the second most common cause of neurodegenerative dementia. Clinically, a previous systematic review reported that DLB accounted for 3.2%–7.1% of all dementia cases based on the results of incidence studies.^1^ Pathologically, 20%–25% of all dementia cases reportedly have a Lewy body pathology.^2^ DLB causes not only cognitive impairment but also physical symptoms, dysautonomia, and neuropsychiatric phenomena. Given that supportive clinical features in the criteria for the clinical diagnosis of DLB include repeated falls,^3^ DLB patients are more likely to experience falls and fall‐related injuries than individuals with normal cognitive functioning and patients with Alzheimer's dementia.^4^, ^5^ More than half of patients with DLB or Parkinson's disease lose the ability to walk independently through hip fracture.^6^ Hence, preventing hip fractures among DLB patients is necessary.

Deficits in multiple cognitive domains, such as low performance in attention and executive function, lead to falls.^7^ Cholinesterase inhibitors (ChEIs), including donepezil, rivastigmine, and galantamine, are reported to be effective as pharmacological treatments for cognitive symptoms in DLB.^3^, ^8^ Memantine, an N‐methyl d‐aspartate receptor antagonist, is also well tolerated and may have benefits, although its efficacy in DLB patients is less clear as compared with that of ChEIs.^9^, ^10^ Although no drug has been approved for the treatment of DLB by the US Food and Drug Administration,^11^ in Japan, Aricept (R), the original brand of donepezil, was approved for suppressing cognitive symptom progression among DLB patients in 2014, and donepezil hydrochloride as a generic was approved in 2019, whereas other ChEIs have not been. It remains unclear whether the use of antidementia drugs is associated with fractures among patients with DLB. We hypothesized that ChEI use is associated with a decreased risk of fractures among DLB patients.

In principle, all Japanese citizens participate in some form of medical insurance. People under age 75 should take part in one of the following health insurance systems: society‐managed, employment‐based health insurance for employees of large companies; public‐corporation‐run health insurance for employees of smaller companies (*Kyokai‐kempo*); mutual aid associations for civil servants and private school teachers (*Kyosai‐kumiai*); seamen's insurance; and National Health Insurance Society for farmers, self‐employed, etc. All people over 75 should take part in a long‐life medical care system (a medical care system for older people in the latter stage of life). The National Database of Health Insurance Claims and Specific Health Checkups of Japan (NDB) is an insurance claim database that collects all of the above‐mentioned health insurance claims. The database includes each patient's individual data, diagnosis, and medications.^12^ We have recently reported that the use of antidementia drugs, such as ChEIs and memantine, was associated with a decreased incidence of hip and major osteoporotic fractures in patients with Alzheimer's dementia using data from the NDB.^13^ In the present study, we further examined the relationship between donepezil use and the incidence of hip and major osteoporotic fractures among DLB patients.

## Methods

### Study population and data sources

The NDB has accumulated all monthly electronic health insurance claims and yearly specific health data of each patient. The data analyzed in this study were as follows: patient's identification number, age, sex, date of consultation for outpatient service and diagnosis, main diagnosis and comorbidities written in a code used in the electronic receipt processing system, and date of procedures and drugs provided to each patient. We used two types of identifiers (IDs 1 and 2, both 64 digits) to link the insurance claims of individual patients and construct the database. ID1 is a hash value generated from the insurer's ID, and the beneficiary's ID, date of birth, and sex. ID2 is a hash value generated from the beneficiary's name, date of birth, and sex. ID1 and ID2 are anonymized variables shown as sequences of alphanumeric characters. NDB data users cannot identify patients using ID1 and ID2. These two IDs allowed us to trace patients' information even when either their ID1 or ID2 changed. In the present study, we used NDB data from fiscal years 2012 to 2018 (April 2012 to March 2019). Among the individuals who were newly registered to have obtained the first medical claim from April 2012 to March 2016 since turning 65 years old, we identified those who met the inclusion and exclusion criteria. The inclusion criteria were as follows: patients (1) were aged ≥65 years at the date of entry; (2) had a look‐back period of 6 months and a follow‐up period of 3 years from the date of registration; (3) had verifiable data receipt throughout the 3.5‐year observation period; and (4) had not been prescribed any antidementia medications in the 6 months before the date of entry. Antidementia drugs were defined as ChEIs (donepezil, rivastigmine, and galantamine) and memantine. The inclusion criterion (2) requires that the analyzed patients were alive during the follow‐up period of 3 years. The inclusion criterion (3) means that the analyzed patients could be traced using either ID1 or ID2. If ID1 and ID2 change simultaneously, the patient cannot be traced. The exclusion criteria were as follows: (1) patients were prescribed antidementia medications but discontinued them for >30 days during the observation period; (2) patients were prescribed antidementia medications for >90 days at one time; (3) patients were newly diagnosed with dementia after the observation period; and (4) patients were prescribed medications that can cause bone fractures (steroids, antidiabetics, antipodagrics, and hormone medications). The exclusion criterion (1) was applied because it is difficult to evaluate the effect of cholinesterase inhibitors on patients who had a discontinuation period because of the low medication possession ratio. The exclusion criterion (2) was applied because the Insurance Review Committee does not approve prescriptions with a >90‐day supply in most cases, and prescription data for >90 days at one time might be a data input error.

The verifiable data included the diagnosis of dementia, osteoporosis, and bone fractures, and the prescription of antidementia or osteoporosis medications. Each drug prescription is assigned a nine‐digit code, and each disease diagnosis is assigned a seven‐digit Japanese Standard Disease Code. The codes used are provided in Tables S1–S5. The entry date of patients with DLB who were prescribed antidementia drugs was referred to as the day of prescribing antidementia drugs for the first time. The entry date of patients without any type of dementia and DLB patients not receiving antidementia drugs was set as the newly registered timing. The observation period for each patient was set at 3 years.

### Data collection

Based on the health insurance claims data, the patients' baseline characteristics, including age, sex, presence of osteoporosis, history of bone mineral density test, fracture history, and prescription of antidementia and osteoporosis drugs, were obtained. The NDB does not include information about fall history. The bone mineral density tests included dual‐energy X‐ray absorptiometry, micro‐densitometry, and ultrasonography. Considering the Japanese guideline for osteoporosis,^14^ we regarded bisphosphonate, parathyroid hormone, denosumab, active vitamin D_3_ compounds, and selective estrogen receptor modulator as osteoporosis drugs.

### Outcomes

DLB patients were classified into the following three groups: (1) received only donepezil among the antidementia drugs (donepezil group); (2) did not receive any antidementia drugs (drug‐naïve group); and (3) received memantine or ChEIs, except donepezil, at least one time. The primary outcome was the incidence rate of hip fracture during the observation period. The secondary outcomes were those of vertebral fracture and major osteoporotic fractures. Major osteoporotic fractures were defined as any hip, vertebral, or radial fracture. When a patient experienced a hip fracture multiple times, we extracted the data of the first fracture owing to the database specification. The same applies to vertebral fracture and radial fracture. We compared the fracture rates between the donepezil and drug‐naïve groups.

### Statistical analyses

First, we compared the characteristics between DLB patients and individuals without dementia. We described the participants' age as 5‐year age groups and the mean value. Next, we conducted one‐to‐one propensity score matching between the donepezil and drug‐naïve groups. We calculated the propensity score using a logistic regression model for receiving donepezil as a function of patient factors, including age, sex, fracture history, osteoporosis, and bone mineral density test before study entry. Osteoporosis was defined as an osteoporosis diagnosis or the use of osteoporosis medications before entry. The fracture risk in people without an osteoporosis diagnosis and medication who underwent a bone mineral density test and that in people without an osteoporosis diagnosis and medication who had never undergone a bone mineral density test are considered to be different. Therefore, we included the bone mineral density test as a matching factor. The C‐statistic was calculated to evaluate the model's discriminatory ability. We conducted nearest‐neighbor matching without replacement, and the caliper was set at 0.2 times the standard deviation of the propensity score estimates. To check the balance, we calculated the standardized differences between the two groups before and after matching. A standardized difference of <0.1 suggests adequate variable balance after propensity score matching.^15^ The fracture rates of the propensity score‐matched patients were compared between the donepezil and drug‐naïve groups. We present numbers and percentages for categorical variables, and means and standard deviations for continuous variables. The Pearson chi‐square test was used for categorical variables, with two‐sided values. The confidence interval of the risk difference was calculated based on the Agresti/Caffo method.^16^ A *P*‐value <0.05 was considered statistically significant. All statistical analyses were conducted in Stata/SE version 17.0 (StataCorp, College Station, TX, USA).

### Ethics approval and consent to participate

The study protocol was approved by the Ministry of Health, Labor, and Welfare, the Institutional Review Board of the Graduate School of Medicine, The University of Tokyo (Approval number: 2020291NI), and the Ethics Committee of the University of Tokyo Hospital. All methods were conducted in accordance with the relevant ethical guidelines and regulations. The study data used were completely anonymous; thus, the need to obtain informed consent from the study participants was waived by the Institutional Review Board of the Graduate School of Medicine, The University of Tokyo.

## Results

**Figure 1:**
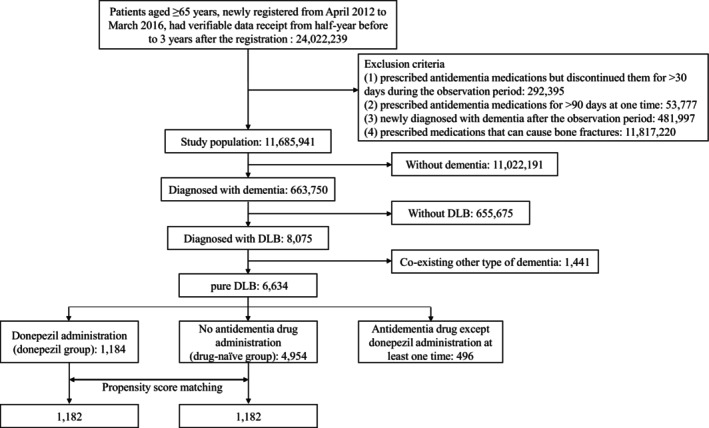
Flow diagram for the patient selection. Flowchart showing how cases and controls were selected from the nationwide health insurance claims database of Japan. Antidementia drugs indicate cholinesterase inhibitors (donepezil, rivastigmine, and galantamine) and memantine. DLB, dementia with Lewy bodies.

The flowchart of patient selection is shown in Figure 1. We identified 24 022 239 people aged ≥65 years who were newly registered from April 2012 to March 2016 and had verifiable data receipts from 6 months before to 3 years after the registration. After excluding people who met at least one of the exclusion criteria, 11 685 941 people were identified. There were 11 022 191 people who had not been diagnosed with dementia, and 663 750 people who had been diagnosed with dementia. Among the 663 750 patients with dementia, 8075 were diagnosed with DLB. People who were also diagnosed with other types of dementia (Alzheimer's dementia, vascular dementia, and frontotemporal lobar degeneration) were excluded, and finally 6634 people were identified as pure‐DLB patients.

Table 1 shows the characteristics of the 11 022 191 people without dementia and the 6634 pure‐DLB patients. The DLB patients were older and were more likely to experience fractures than people without dementia. The proportion of women was higher in DLB patients. DLB patients were more frequently diagnosed with osteoporosis or prescribed osteoporosis medications.

**Table 1:** Characteristics of cognitively healthy people and DLB patients

		People without dementia (*n* = 11 022 191)		DLB patients (*n* = 6634)		
Patient group		*n*	%	*n*	%	Standardized difference
Age group	65–69	3 371 026	30.6	647	9.8	0.853
	70–74	3 895 225	35.3	1317	19.9	
	75–79	1 910 252	17.3	1541	23.2	
	80–84	1 113 993	10.1	1598	24.1	
	85–89	524 535	4.8	1084	16.3	
	90+	207 160	1.9	447	6.7	
Age (mean ± SD)		73.3 ± 6.3		78.9 ± 7.1		−0.840
Male		4 010 598	36.4	1896	28.6	−0.167
Fracture history		68 861	0.6	130	2.0	−0.118
Osteoporosis		1 452 109	13.2	1185	17.9	−0.130
Bone mineral density test		823 272	7.5	531	8.0	−0.020

Fracture history was defined as major osteoporotic fractures in the 6‐month look‐back period. Osteoporosis was defined as osteoporosis diagnosis or osteoporosis medication use in the 6‐month look‐back period. Bone mineral density test indicates people who had undergone any bone mineral density test in the 6‐month look‐back period.

DLB, dementia with Lewy bodies; SD, standard deviation.

Among the 6634 DLB patients, 1184 people were prescribed with donepezil only (donepezil group), and 496 people were prescribed with antidementia drugs, except donepezil. The numbers of individuals who received galantamine, rivastigmine, or memantine among those 496 people are shown in Figure S1. Altogether, 4954 people were not prescribed any antidementia drugs (drug‐naïve group). We compared the characteristics of the donepezil group with those of the drug‐naïve group. Based on our previous report on Alzheimer's dementia and antidementia drugs,^13^ we conducted one‐to‐one propensity score matching. The C‐statistic for the logistic regression was 0.586. The propensity score matching yielded 1182 pairs. Table 2 shows the characteristics before and after matching. Before the propensity score matching, DLB patients with donepezil were older than the drug‐naïve patients. The proportion of patients with osteoporosis diagnosis or medications and that of patients who had undergone a bone mineral density test before entry were higher in the donepezil group. After the propensity score matching, the standardized differences were all <0.1, indicating well‐balanced distributions of the patient characteristics.

**Table 2:** Baseline characteristics of DLB patients without antidementia drugs and with donepezil

		Before propensity score matching					After propensity score matching				
		Without antidementia drugs (*n* = 4954)		With donepezil (*n* = 1184)			Without antidementia drugs (*n* = 1182)		With donepezil (*n* = 1182)		
Unadjusted or Adjusted		*n*	%	*n*	%	Standardized difference	*n*	%	*n*	%	Standardized difference
Age	65–69	517	10.4	95	8.0	−0.126	98	8.3	95	8.0	0.003
	70–74	1060	21.4	174	14.7		174	14.7	174	14.7	
	75–79	1099	22.2	304	25.7		300	25.4	304	25.7	
	80–84	1140	23.0	331	28.0		327	27.7	331	28.0	
	85–89	791	16.0	204	17.2		205	17.3	204	17.3	
	90+	347	7.0	76	6.4		78	6.6	74	6.3	
Age (mean ± SD)		78.7 ± 7.2		79.6 ± 6.6		−0.120	79.5 ± 6.6		79.5 ± 6.6		<0.001
Male		1357	27.4	384	32.4	0.110	369	31.2	382	32.3	0.024
Fracture history		93	1.9	22	1.9	0.001	12	1.0	22	1.9	−0.071
Osteoporosis		792	16.0	287	24.2	−0.207	291	24.6	285	24.1	0.012
Bone mineral density test		344	6.9	137	11.6	−0.160	138	11.7	136	11.5	0.005

Fracture history was defined as major osteoporotic fractures in the 6‐month look‐back period. Osteoporosis was defined as osteoporosis diagnosis or osteoporosis medication use in the 6‐month look‐back period. Bone mineral density test indicates people who had undergone any bone mineral density test in the 6‐month look‐back period.

DLB, dementia with Lewy bodies; SD, standard deviation.

The fracture cases for 3 years and the incidence rates are shown in Table 3. The incidence rate of hip fracture was significantly lower in the donepezil group than in the drug‐naïve group (0.60 vs. 1.44/100 person‐years, *P* < 0.001), whereas those of vertebral and major osteoporotic fractures were the same between the two groups. According to the rules for publication of NDB data, we did not show the number of radial fracture alone, which is <10.

**Table 3:** Fractures over 3 years in the DLB patients without antidementia drugs and with donepezil after propensity score matching

Outcome		Without antidementia drugs	With donepezil	Incidence rate ratio, 95% CI	Incidence rate difference, 95% CI (/100 person‐years)	*P*‐value
Hip fracture	*n* (%)	50 (4.2)	21 (1.8)	0.42 [0.24, 0.70]	−0.84 [−1.31, −0.37]	<0.001
	Incidence rate (/100 person‐years)	1.44	0.60			
Vertebral fracture	*n* (%)	95 (8.0)	105 (8.9)	1.12 [0.84, 1.49]	0.32 [−0.49, 1.14]	0.44
	Incidence rate (/100 person‐years)	2.78	3.11			
Major osteoporotic fractures	*n* (%)	142 (12.0)	126 (10.7)	0.88 [0.69, 1.13]	−0.49 [−1.45, 0.47]	0.32
	Incidence rate (/100 person‐years)	4.25	3.76			

We analyzed 1182 propensity score‐matched pairs. Major osteoporotic fractures indicate any of hip fracture, vertebral fracture, and radius fracture. The observation period after a fracture happened was excluded when calculating person‐years.

CI, confidence interval; DLB, dementia with Lewy bodies.

## Discussion

In the present study, we examined the association between donepezil use and bone fracture risk in DLB patients using a Japanese nationwide database. Almost all Japanese people take part in some public health insurance system, with the exception of people requiring public assistance owing to poverty, and the database covers about 95% of all administrative claims data in Japan. The analysis identified 6634 pure‐DLB patients aged ≥65 years and revealed that donepezil use was associated with a decreased risk of hip fracture by comparing the data of 1182 propensity score‐matched pairs. A total of 1184 out of 6634 patients (17.8%) with pure DLB had received donepezil for 3 years. The low prescription rate might be because donepezil treatment for DLB was not approved in Japan before September 2014. In contrast to the case for hip fractures, the incidence rate of vertebral fracture was the same between DLB patients receiving donepezil and those not receiving antidementia drugs. This might be because about half of vertebral fractures are reported to occur without a fall or some other kind of trauma, while most hip fractures occur with a fall or other trauma.^17^

Although there are no reports about the association between ChEIs and fractures in DLB patients, several studies have reported on the association between ChEIs and fracture risk among overall dementia or Alzheimer's dementia cases. ChEI use has been reported to be associated with a clinically important reduction in fracture risk in male veterans with dementia.^18^ Some case–control studies and our previous study showed that ChEI use was associated with a decreased risk of fractures in older people with Alzheimer's dementia.^13^, ^19^, ^20^ Both DLB and Parkinson's disease are α‐synucleinopathies with motor symptoms such as freezing of gait and postural instability. A small trial showed that the use of donepezil reduced the fall frequency, and a randomized controlled trial showed that rivastigmine improved the gait stability of patients with Parkinson's disease.^21^, ^22^ A meta‐analysis of randomized control trials in 2011 reported that ChEI use was not associated with falls and fractures compared with a placebo in patients with cognitive impairment.^23^ On the other hand, another meta‐analysis in 2023 showed that ChEI use was associated with a reduced risk of falls compared with placebo in patients with cognitive impairment.^24^ Furthermore, there was a trend between ChEIs and reduced risk of falls compared with placebo in patients with Parkinson disease or DLB, although the difference was not statistically significant.^24^ This trend supports our study. In the present study, we focused on donepezil hydrochloride. It was difficult to evaluate the relationship between other antidementia drugs (galantamine, rivastigmine, and memantine) and fractures because only a few patients receiving these drugs got fractures.

The potential reason why DLB patients are more likely to fall is because they often have autonomic, motor, and visuospatial impairments together with impaired judgment, executive function, and fluctuating cognition.^25^ Considering the findings of several reports, including those mentioned above, our study results may be explained as follows. First, the benefits of ChEIs on DLB and Alzheimer's dementia include improvements in cognitive function and global assessment.^3^, ^8^, ^26^ Additionally, ChEIs have a beneficial effect on hallucinations and delusions in DLB.^3^, ^8^ These effects might decrease the risk of falls in DLB patients in terms of cognitive function and neuropsychiatric symptoms. Second, as discussed above, ChEIs might alleviate postural instability and gait difficulty among DLB patients. These effects prevent falls, as they improve motor function, although we could not elucidate whether donepezil use was associated with falls in our study. Third, donepezil itself may ameliorate osteoporosis because donepezil prevents bone loss by inhibition of osteoclast differentiation.^27^

Whereas this study focused on the positive effects of ChEIs, it is important to consider adverse effects as well as positive effects. The major adverse effects of ChEIs are bradycardia and syncope, which lead to falls and fractures. A cohort study targeted at overall dementia showed that use of ChEIs was associated with increased rates of bradycardia, syncope, and hip fracture.^28^ The results in our study may have been obtained because the above‐mentioned positive effects for patients with DLB were greater than such adverse effects.

There are several limitations to this study. First, the diagnosis might not be based on the DLB Consortium criteria,^3^ and the diagnostic criteria might differ among clinicians because NDB is a medical claim database. Second, while we adjusted for age, sex, fracture history, osteoporosis, and bone mineral density test before entry, confounding factors related to fractures, such as walking ability, hypnotics, alcohol intake, family history of fracture, are still present.^29^, ^30^ The NDB does not include information about each patient's activities of daily living, lifestyle, and family history. Third, there is a selection bias in the present study. Patients who did not respond to donepezil might be likely to discontinue the pharmacological treatment, and we excluded those patients who discontinued the use of antidementia drugs for >30 days during the observation period. Therefore, the positive effects of donepezil might be overestimated. The risk for all‐cause mortality is reported to increase after hip fracture,^31^ and the inclusion criterion that requires patients to be alive during 3 years might also introduce a selection bias.

Several strategies for fall prevention in older DLB patients exist, including medical management of Parkinsonism, cognitive strategies, managing orthostatic hypotension, and physiotherapy. Some treatments have been suggested to reduce falls in patients with Parkinson's disease.^25^ However, no interventions except osteoporosis treatment have been proved to reduce the risk of fractures in DLB patients. There are no curative therapies for DLB, and treatments to maintain the activities of daily living are required. The present study has elucidated a new benefit of donepezil in DLB.

In conclusion, our study data revealed that donepezil administration was significantly associated with a decreased risk of hip fracture among Japanese patients with DLB. Patients with DLB are likely to fall repeatedly. It is worth considering the prescription of donepezil for DLB patients.

## Disclosure statement

The authors declare no conflict of interest.

## Supporting information


**Data S1.** Supporting Information.

## Data Availability

The data that support the findings of this study are available from contracts with the hospitals providing data to the database. Restrictions apply to the availability of these data, which were used under license for this study. Data are available from the author(s) with the permission of contracts with the hospitals providing data to the database.
