# Supplementary material for: Relationship between donepezil and fracture risk in patients with dementia with Lewy bodies
Source: Geriatr Gerontol Int. 2024 Jun 26;24(8):782–8. doi: 10.1111/ggi.14929 (PMC11503602; doi:10.1111/ggi.14929)
Supplement: Supplementary file 1 — Data S1. Supporting Information. [file GGI-24-782-s001.docx]

**Supplemental Materials**

**Table of Contents**

**Table S1. Ministry of Health, Labor and Welfare standard code for diagnosis of disease; Osteoporosis……….…………………………………………………………. 2**

**Table S2. Ministry of Health, Labor and Welfare standard code for diagnosis of disease; Osteoporotic fragile fractures………………………………………………. 5**

**Table S3. Ministry of Health, Labor and Welfare standard code for diagnosis of disease; Dementia……………………………………………………………………... 7**

**Table S4. Ministry of Health, Labor and Welfare standard code for prescription of drugs; Osteoporosis drugs……………………………………………………………. 9**

**Table S5. Ministry of Health, Labor and Welfare standard code for prescription of drugs; Antidementia drugs………………………………………………………….. 16**

**Figure S1. The numbers of individuals who received each antidementia drug among DLB patients who received memantine or ChEIs, except donepezil, at least one time………………………………………………………………………………22**

**Table S1. Ministry of Health, Labor and Welfare standard code for diagnosis of disease; Osteoporosis**

| Code | English description | ICD10 | ICD-10 category | ICD-10 description |
| --- | --- | --- | --- | --- |
| 7330006 | Osteoporosis | M8199 | Osteoporosis, unspecified | M819* |
| 8844204 | Osteoporosis with pathological pelvis fracture | M8095 | Unspecified osteoporosis with pathological fracture | M809* |
| 8844205 | Osteoporosis with pathological vertebral fracture | M8098 | Unspecified osteoporosis with pathological fracture | M809* |
| 8844206 | Osteoporosis with pathological radius fracture | M8093 | Unspecified osteoporosis with pathological fracture | M809* |
| 8844207 | Osteoporosis with pathological multiple fracture | M8090 | Unspecified osteoporosis with pathological fracture | M809* |
| 8844208 | Osteoporosis with pathlogical femoral fracture | M8095 | Unspecified osteoporosis with pathological fracture | M809* |
| 8844209 | Osteoporosis with pathological fracture | M809 | Unspecified osteoporosis with pathological fracture | M809 |
| 7330024 | Senile Osteoprosis | M8189 | Senile osteoporosis | M818* |
| 8848170 | Senile Osteoporosis with pathological pelvis fracture | M8085 | Other osteoporosis with pathological fracture | M808* |
| 8848171 | Senile Osteoporosis with pathological vertebral fracture | M8088 | Other osteoporosis with pathological fracture | M808* |
| 8848172 | Senile Osteoporosis with pathological radius fracture | M8083 | Other osteoporosis with pathological fracture | M808* |
| 8848173 | Senile Osteoporosis with pathological multiple fracture | M8080 | Other osteoporosis with pathological fracture | M808* |
| 8848174 | Senile Osteoporosis with pathological femoral fracture | M8085 | Other osteoporosis with pathological fracture | M808* |
| 8844345 | Senile Osteoporosis with pathological fracture | M808 | Other osteoporosis with pathological fracture | M808 |
| 7330020 | Cervical Osteoporosis | M8168 | Localized osteoporosis [Lequesne] | M816* |
| 8844167 | Cervical Osteoporosis with pathological fracture | M8098 | Unspecified osteoporosis with pathological fracture | M809* |
| 8849529 | Osteoporosis with high risk of fracture | M8099 | Unspecified osteoporosis with pathological fracture | M809* |
| 7330023 | Juvinile Osteoporosis | M8159 | Idiopathic osteoporosis | M815* |
| 8844237 | Juvinile Osteoporosis with phathlogical fracture | M805 | Idiopathic osteoporosis with pathological fracture | M805 |
| 8849536 | Severe Osteoporosis | M8099 | Unspecified osteoporosis with pathological fracture | M809* |
| 8835331 | Postoperatie malabsorptive Osteoporosis | M8139 | Postsurgical malabsorption osteoporosis | M813* |
| 8844238 | Postoperatie malabsorptive Osteoporosis with pathological fracture | M803 | Postsurgical malabsorption osteoporosis with pathological fracture | M803 |
| 7330026 | Glucocorticoid induced osteoporosis | M8149 | Drug-induced osteoporosis | M814* |
| 8844272 | Glucocorticoid induced osteoporosis with pathological fracres | M804 | Drug-induced osteoporosis with pathological fracture | M804 |
| 7330013 | Spine osteoporosis | M8168 | Localized osteoporosis [Lequesne] | M816* |
| 8844274 | Spine osteoporosis with pathological fracture | M8098 | Unspecified osteoporosis with pathological fracture | M809* |
| 8844302 | Idiopathic Osteoporosis | M8159 | Idiopathic osteoporosis | M815* |
| 8844303 | Idiopathic Osteoporosis with pathological fracture | M805 | Idiopathic osteoporosis with pathological fracture | M805 |
| 8848091 | Idiopathic Juvinile Osteoporosis | M815 | Postoophorectomy osteoporosis | M811* |
| 7330029 | Secondary Osteoporosis | M8199 | Osteoporosis, unspecified | M819* |
| 8844308 | Secondary Osteoporosis with pathological fracture | M809 | Unspecified osteoporosis with pathological fracture | M809 |
| 8838912 | Disuse Osteoporosis | M8129 | Osteoporosis of disuse | M812* |
| 8844313 | Disuse Osteoporosis with pathological fracture | M802 | Osteoporosis of disuse with pathological fracture | M802 |
| 7330028 | Postmenopausal osteoporosis | M8109 | Postmenopausal osteoporosis | M810* |
| 8844319 | Postmenopausal osteoporosis with pathological pelvis fracture | M8005 | Postmenopausal osteoporosis with pathological fracture | M800* |
| 8844320 | Postmenopausal osteoporosis with pathological vertebral fracture | M8008 | Postmenopausal osteoporosis with pathological fracture | M800* |
| 8844321 | Postmenopausal osteoporosis with pathological radius fracture | M8003 | Postmenopausal osteoporosis with pathological fracture | M800* |
| 8844322 | Postmenopausal osteoporosis with pathological multiple fracture | M8000 | Postmenopausal osteoporosis with pathological fracture | M800* |
| 8844323 | Postmenopausal osteoporosis with pathological femur fracture | M8005 | Postmenopausal osteoporosis with pathological fracture | M800* |
| 8844324 | Postmenopausal osteoporosis with pathological fracture | M800 | Postmenopausal osteoporosis with pathological fracture | M800* |
| 8840727 | Drug-induced osteoporosis | M8149 | Drug-induced osteoporosis | M814* |
| 8844332 | Drug-induced osteoporosis with pathological fracture | M804 | Drug-induced osteoporosis with pathological fracture | M804 |
| 8840913 | Ovarectomized osteoporosis | M8119 | Postoophorectomy osteoporosis | M811* |
| 8844338 | Ovarectomized osteoporosis with pathological fracture | M801 | Postoophorectomy osteoporosis with pathological fracture | M801 |

**Table S2. Ministry of Health, Labor and Welfare standard code for diagnosis of disease; Osteoporotic fragile fractures**

| **Code** | **English description** | **ICD10** | **ICD-10 description** | **ICD-10 category** |
| --- | --- | --- | --- | --- |
| 8833324 | colles fracture | S5250 | Fracture of radius | S52.5* |
| 8208004 | hip fracture | S7200 | Fracture of femur | S72.0* |
| 8029005 | Fracture of unspecified body region | T1420 | unspecified body region | T14.2* |
| 8842076 | Steroid-induced vertebral compression fracture | M8048 | Fracture of spine | M80.4* |
| 8058002 | Fracture of spine, level unspecified | T08-0 | Fracture of spine | T08.-* |
| 8058003 | Fracture of spine, level unspecified | T08-0 | Fracture of spine | T08.-* |
| 7331021 | Other disorders of continuity of bone | M8448 | Fracture of spine | M84.4* |
| 8848369 | Pathological fracture, not elsewhere classified | M8449 | unspecified body region | M84.4* |
| 8290022 | Multiple fractures, unspecified | T0290 | unspecified body region | T02.9* |
| 8837211 | Pathological fracture, not elsewhere classified | M8448 | Fracture of neck | M84.4* |
| 8837243 | Pathological fracture, not elsewhere classified | M8448 | Fracture of spine | M84.4* |
| 8837252 | Pathological fracture, not elsewhere classified | M8448 | Fracture of spine | M84.4* |
| 8837257 | Pathological fracture, not elsewhere classified | M8448 | Fracture of spine | M84.4* |
| 8837260 | Pathological fracture, not elsewhere classified | M8448 | Fracture of spine | M84.4* |
| 8837298 | Femoral neck lateral penetrating fracture | S7200 | Fracture of femur | S72.0* |
| 8837299 | Lateral femoral neck fracture | S7200 | Fracture of femur | S72.0* |
| 8208009 | femoral neck fracture | S7200 | Fracture of femur | S72.0* |
| 8837303 | Medial femoral neck fracture | S7200 | Fracture of femur | S72.0* |
| 8210002 | femoral fracture | S7290 | Fracture of femur | S72.9* |
| 8837305 | femoral subhead fracture | S7200 | Fracture of femur | S72.0* |
| 8837306 | Medial femoral head fracture | S7200 | Fracture of femur | S72.0* |
| 8837311 | femoral neck fracture | S7200 | Fracture of femur | S72.0* |
| 8837312 | Multiple Femoral Fractures | S7270 | Fracture of femur | S72.7* |
| 8837314 | femoral trochanteric fracture | S7210 | Fracture of femur | S72.1* |
| 8837320 | femoral insufficiency fracture | S7290 | Fracture of femur | S72.9* |
| 8837321 | femoral comminuted fracture | S7290 | Fracture of femur | S72.9* |
| 8837382 | greater trochanteric fracture | S7210 | Fracture of femur | S72.1* |
| 8841997 | Sequelae of fracture of spine | T911 | Fracture of spine | T91.1* |
| 8209001 | Sequelae of fracture of femur | T931 | Fracture of femur | T93.3* |
| 8058012 | Sequelae of fracture of spine | T911 | Fracture of spine | T91.1* |
| 8842012 | Sequelae of fracture of radius | T921 | Fracture of radius | T92.2* |
| 8841998 | Sequelae of fracture of spine | T911 | Fracture of spine | T91.1* |
| 8058006 | vertebral fracture | T08-0 | Fracture of spine | T08.-* |
| 8058007 | vertebral fracture | T08-0 | Fracture of spine | T08.-* |
| 8838017 | Distal radius fracture | S5250 | Fracture of radius | S52.5* |
| 8130016 | radius fracture | S5280 | Fracture of radius | S52.8* |
| 8848984 | Radial ulnar distal fracture | S5260 | Fracture of radius | S52.6* |
| 8838026 | Fracture of shafts of both ulna and radius | S5240 | Fracture of radius | S52.4* |
| 8054003 | Lumbar compression fracture | S3200 | Fracture of spine | S32.0* |
| 8054016 | Lumbar fracture | S3200 | Fracture of spine | S32.0* |
| 8845617 | Multiple compression fractures of the lumbar vertebrae | S3270 | Fracture of spine | S32.7* |
| 8845618 | Multiple fractures of the lumbar spine | S3270 | Fracture of spine | S32.7* |
| 8844926 | lumbar vertebral body fracture | S3200 | Fracture of spine | S32.0* |

**Table S3. Ministry of Health, Labor and Welfare standard code for diagnosis of disease; Dementia**

| Code | English description | ICD10 | ICD-10 description | ICD-10 category |
| --- | --- | --- | --- | --- |
| 8842547 | Alcoholic dementia | F107 | Mental and behavioural disorders due to use of alcohol | F10* |
| 8842548 | Presenile dementia of Alzheimer's type | G300 | Alzheimer disease with early onset | G30* |
| 8842549 | Alzheimer's dementia | G309 | Alzheimer disease, unspecified | G30* |
| 8842550 | Aheimer's atypical dementia | G308 | Other Alzheimer disease | G30* |
| 8842551 | seniel dementia of Alzheimer's type | G301 | Alzheimer disease with late onset | G30* |
| 3310002 | Alzheimer's disease | G309 | Alzheimer disease, unspecified | G30* |
| 8842565 | Vascular dementia f acute onset | F010 | Vascular dementia of acute onset | F01* |
| 8842571 | vascular dementia | F019 | Vascular dementia, unspecified | F01* |
| 8842575 | primary dementia | F03 | Unspecified dementia | F03* |
| 8848534 | argyrophilic franular dementia | G238 | Other specified degenerative diseases of basal ganglia | G23* |
| 8842591 | presenile dementia | F03 | Unspecified dementia | F03* |
| 8844891 | frontomemporal dementia | G310 | Circumscribed brain atrophy - Frontotemporal dementia (FTD) | G31* |
| 8842608 | multi-infarct dementia | F011 | Multi-infarct dementia | F01* |
| 8842615 | secondary dementia | F03 | Unspecified dementia | F03* |
| 8842618 | dementia | F03 | Unspecified dementia | F03* |
| 8842619 | delirium superinposed on dementia | F051 | Delirium superimposed on dementia | F05* |
| 8842620 | delirium not superinposed on dementia | F050 | Delirium not superimposed on dementia, so described | F05* |
| 8842621 | dementia in cerebral lipidosis | E756 | Lipid storage disorder, unspecified | E75* |
| 8845600 | dementia in huntington's desease | G10 | Huntington disease | G10* |
| 8845602 | parkinson's desease dementia | F023 | Dementia in Parkinson disease (G20†) | F02* |
| 8842625 | subcortical dementia | F012 | Subcortical vascular dementia | F01* |
| 8842626 | cortical dementia | F011 | Multi-infarct dementia | F01* |
| 8845840 | dementia with Lewy bodies | G318 | Other specified degenerative diseases of nervous system | G31* |
| 8842637 | senile dementia | F03 | Unspecified dementia | F03* |
| 8842638 | senile dementia delusional type | F03 | Unspecified dementia | F03* |
| 8842639 | senile dementia dpressice type | F03 | Unspecified dementia | F03* |
| 8845516 | demntia of HIV infection | B220 | HIV disease resulting in encephalopathy | B22* |
| 8846103 | ramilial creutzfeldt-jakob disease | A810 | Creutzfeldt-Jakob disease | A81* |
| 0461004 | Creutzfeldt-Jakob disease | F021 | Dementia in Creutzfeldt-Jakob disease (A81.0†) | F02* |
| 8846131 | sporadic creutzfeldt-jakob desase | A810 | Creutzfeldt-Jakob disease | A81* |
| 8847912 | CADASIL | F011 | Multi-infarct dementia | F01* |
| 8847913 | CARASIL | F011 | Multi-infarct dementia | F01* |
| 2902006 | geriatric depression | F03 | Unspecified dementia | F03* |
| 2902008 | senile delusions | F03 | Unspecified dementia | F03* |
| 2902012 | senile psychosis | F03 | Unspecified dementia | F03* |

**Table S4. Ministry of Health, Labor and Welfare standard code for prescription of drugs; Osteoporosis drugs**

| **Code** | **ATC codes** | **Drug Name** | **General Name** |
| --- | --- | --- | --- |
| 610451020 | M05BA04 | Fosamac tablet 5mg | Alendronate Sodium Hydrate |
| 610451021 | M05BA04 | Bonalon tablet 5mg | Alendronate Sodium Hydrate |
| 610462001 | M05BA07 | Actonel tablet 2.5mg | Sodium Risedronate Hydrate |
| 610462003 | M05BA07 | Benet tablet 2.5mg | Sodium Risedronate Hydrate |
| 613990084 | M05BA01 | Didronel Tablets 200mg | Etidronate Disodium |
| 620004357 | M05BA04 | Fosamac tablet 35mg | Alendronate Sodium Hydrate |
| 620004359 | M05BA04 | Bonalon tablet 35mg | Alendronate Sodium Hydrate |
| 620004865 | M05BA07 | Actonel tablet 17.5mg | Sodium Risedronate Hydrate |
| 620004871 | M05BA07 | Benet tablet 17.5mg | Sodium Risedronate Hydrate |
| 620009099 | － | Bonoteo tablet 1mg | Minodronic Acid Hydrate |
| 620009100 | － | Recarbon tablet 1mg | Minodronic Acid Hydrate |
| 620009303 | M05BA04 | Alendronate tablet 5mg | Alendronate Sodium Hydrate |
| 620009305 | M05BA04 | Alendronate tablet 5mg | Alendronate Sodium Hydrate |
| 621896801 | M05BA04 | Alendronate tablet 5mg | Alendronate Sodium Hydrate |
| 621977502 | M05BA04 | Alendronate tablet 5mg | Alendronate Sodium Hydrate |
| 622041601 | M05BA07 | Risedronate tablet 2.5mg | Sodium Risedronate Hydrate |
| 622045601 | M05BA07 | Risedronate tablet 2.5mg | Sodium Risedronate Hydrate |
| 622050601 | M05BA07 | Risedronate tablet 2.5mg | Sodium Risedronate Hydrate |
| 622053501 | M05BA07 | Risedronate tablet 2.5mg | Sodium Risedronate Hydrate |
| 622053701 | M05BA07 | Risedronate tablet 2.5mg | Sodium Risedronate Hydrate |
| 622058101 | M05BA07 | Risedronate tablet 2.5mg | Sodium Risedronate Hydrate |
| 622061801 | M05BA07 | Risedronate tablet 2.5mg | Sodium Risedronate Hydrate |
| 622063401 | M05BA07 | Risedronate tablet 2.5mg | Sodium Risedronate Hydrate |
| 622091301 | M05BA04 | Alendronate tablet 5mg | Alendronate Sodium Hydrate |
| 622091401 | M05BA04 | Alendronate tablet 35mg | Alendronate Sodium Hydrate |
| 622093101 | M05BA07 | Minodoronate tablet 1mg | Minodronic Acid Hydrate |
| 622094601 | － | Recarbon tablet 50mg | Minodronic Acid Hydrate |
| 622097202 | － | Risedronate tablet 2.5mg | Sodium Risedronate Hydrate |
| 622101602 | M05BA04 | Alendronate tablet 35mg | Alendronate Sodium Hydrate |
| 622110601 | M05BA04 | Alendronate tablet 35mg | Alendronate Sodium Hydrate |
| 622110701 | M05BA04 | Alendronate tablet 35mg | Alendronate Sodium Hydrate |
| 622110801 | M05BA07 | Risedronate tablet 2.5mg | Sodium Risedronate Hydrate |
| 622113503 | M05BA04 | Alendronate tablet 35mg | Alendronate Sodium Hydrate |
| 622118001 | M05BA07 | Risedronate tablet 2.5mg | Sodium Risedronate Hydrate |
| 622118301 | M05BA04 | Alendronate tablet 35mg | Alendronate Sodium Hydrate |
| 622121501 | M05BA04 | Alendronate tablet 5mg | Alendronate Sodium Hydrate |
| 622121601 | M05BA04 | Alendronate tablet 35mg | Alendronate Sodium Hydrate |
| 622122401 | M05BA07 | Risedronate tablet 2.5mg | Sodium Risedronate Hydrate |
| 622122501 | M05BA04 | Alendronate tablet 35mg | Alendronate Sodium Hydrate |
| 622124401 | M05BA07 | Risedronate tablet 2.5mg | Sodium Risedronate Hydrate |
| 622127901 | M05BA04 | Alendronate tablet 5mg | Alendronate Sodium Hydrate |
| 622128001 | M05BA04 | Alendronate tablet 35mg | Alendronate Sodium Hydrate |
| 622130101 | M05BA07 | Risedronate tablet 2.5mg | Sodium Risedronate Hydrate |
| 622147701 | M05BA07 | Risedronate tablet 2.5mg | Sodium Risedronate Hydrate |
| 622159101 | M05BA07 | Risedronate tablet 2.5mg | Sodium Risedronate Hydrate |
| 622186801 | M05BA04 | Alendronate tablet 5mg | Alendronate Sodium Hydrate |
| 622186901 | M05BA04 | Alendronate tablet 35mg | Alendronate Sodium Hydrate |
| 622188801 | M05BA07 | Risedronate tablet 2.5mg | Sodium Risedronate Hydrate |
| 622205001 | M05BA07 | Risedronate tablet 2.5mg | Sodium Risedronate Hydrate |
| 622206401 | M05BA07 | Risedronate tablet 17.5mg | Sodium Risedronate Hydrate |
| 622219901 | M05BA07 | Risedronate tablet 17.5mg | Sodium Risedronate Hydrate |
| 622222101 | M05BA04 | Alendronate tablet 5mg | Alendronate Sodium Hydrate |
| 622222201 | M05BA04 | Alendronate tablet 35mg | Alendronate Sodium Hydrate |
| 622223501 | M05BA04 | Alendronate Oral Jerry 35mg | Alendronate Sodium Hydrate |
| 622224701 | M05BA07 | Benet tablet 75mg | Sodium Risedronate Hydrate |
| 622226001 | M05BA07 | Actonel tablet 75mg | Sodium Risedronate Hydrate |
| 622227101 | M05BA07 | Risedronate tablet 17.5mg | Sodium Risedronate Hydrate |
| 622228901 | M05BA07 | Risedronate tablet 17.5mg | Sodium Risedronate Hydrate |
| 622229301 | M05BA07 | Risedronate tablet 17.5mg | Sodium Risedronate Hydrate |
| 622233301 | M05BA07 | Risedronate tablet 17.5mg | Sodium Risedronate Hydrate |
| 622235101 | M05BA07 | Risedronate tablet 2.5mg | Sodium Risedronate Hydrate |
| 622235201 | M05BA07 | Risedronate tablet 17.5mg | Sodium Risedronate Hydrate |
| 622236401 | M05BA07 | Risedronate tablet 17.5mg | Sodium Risedronate Hydrate |
| 622238401 | M05BA07 | Risedronate tablet 17.5mg | Sodium Risedronate Hydrate |
| 622239001 | M05BA07 | Risedronate tablet 17.5mg | Sodium Risedronate Hydrate |
| 622241501 | M05BA07 | Risedronate tablet 17.5mg | Sodium Risedronate Hydrate |
| 622243601 | M05BA07 | Risedronate tablet 17.5mg | Sodium Risedronate Hydrate |
| 622245401 | M05BA07 | Risedronate tablet 17.5mg | Sodium Risedronate Hydrate |
| 622245901 | M05BA07 | Risedronate tablet 2.5mg | Sodium Risedronate Hydrate |
| 622246001 | M05BA07 | Risedronate tablet 17.5mg | Sodium Risedronate Hydrate |
| 622247201 | M05BA07 | Risedronate tablet 17.5mg | Sodium Risedronate Hydrate |
| 622248801 | M05BA07 | Risedronate tablet 17.5mg | Sodium Risedronate Hydrate |
| 622252601 | M05BA07 | Risedronate tablet 17.5mg | Sodium Risedronate Hydrate |
| 622255801 | M05BA04 | Alendronate tablet 5mg | Alendronate Sodium Hydrate |
| 622255901 | M05BA04 | Alendronate tablet 35mg | Alendronate Sodium Hydrate |
| 622257301 | M05BA07 | Risedronate tablet 17.5mg | Sodium Risedronate Hydrate |
| 622258401 | M05BA07 | Risedronate tablet 17.5mg | Sodium Risedronate Hydrate |
| 622259901 | M05BA07 | Risedronate tablet 17.5mg | Sodium Risedronate Hydrate |
| 622260501 | M05BA07 | Risedronate tablet 17.5mg | Sodium Risedronate Hydrate |
| 622260601 | M05BA07 | Risedronate tablet 17.5mg | Sodium Risedronate Hydrate |
| 622291001 | M05BA04 | Alendronate tablet 5mg | Alendronate Sodium Hydrate |
| 622291101 | M05BA04 | Alendronate tablet 35mg | Alendronate Sodium Hydrate |
| 622471501 | M05BA06 | Bonviba 100mg | Ibandronate Sodium Hydrate |
| 622532001 | M05BA04 | Alendronate tablet 5mg | Alendronate Sodium Hydrate |
| 622532101 | M05BA04 | Alendronate tablet 35mg | Alendronate Sodium Hydrate |
| 622617400 | M05BA07 | Risedronate tablet 17.5mg | Sodium Risedronate Hydrate |
| 622621101 | － | Minodoronate tablet 1mg | Minodronic Acid Hydrate |
| 622621201 | － | Minodoronate tablet 50mg | Minodronic Acid Hydrate |
| 622627301 | － | Minodoronate tablet 1mg | Minodronic Acid Hydrate |
| 622627401 | － | Minodoronate tablet 50mg | Minodronic Acid Hydrate |
| 622630301 | － | Minodoronate tablet 1mg | Minodronic Acid Hydrate |
| 622630401 | － | Minodoronate tablet 50mg | Minodronic Acid Hydrate |
| 622634301 | － | Minodoronate tablet 1mg | Minodronic Acid Hydrate |
| 622634401 | － | Minodoronate tablet 50mg | Minodronic Acid Hydrate |
| 622635701 | － | Minodoronate tablet 1mg | Minodronic Acid Hydrate |
| 622635801 | － | Minodoronate tablet 50mg | Minodronic Acid Hydrate |
| 622637301 | － | Minodoronate tablet 50mg | Minodronic Acid Hydrate |
| 622637401 | － | Minodoronate tablet 1mg | Minodronic Acid Hydrate |
| 622639001 | － | Minodoronate tablet 1mg | Minodronic Acid Hydrate |
| 622639101 | － | Minodoronate tablet 50mg | Minodronic Acid Hydrate |
| 622640401 | － | Minodoronate tablet 1mg | Minodronic Acid Hydrate |
| 622640501 | － | Minodoronate tablet 50mg | Minodronic Acid Hydrate |
| 622643701 | － | Minodoronate tablet 1mg | Minodronic Acid Hydrate |
| 622643801 | － | Minodoronate tablet 50mg | Minodronic Acid Hydrate |
| 622143701 |  | Bonalon Injection 1mg | Alendronate Sodium Hydrate |
| 622255201 | M05BA06 | Bonviva Intravenous Injection | Ibandronate Sodium Hydrate |
| 622538501 | M05BA04 | Alendronate injection 1mg | Alendronate Sodium Hydrate |
| 622545801 | M05BA04 | Alendronate injection 1mg | Alendronate Sodium Hydrate |
| 622518601 | M05BA08 | Reclast Injection 5mg | Zoledronic Acid Hydrate |
| 622239101 | M05BX04 | PRALIA SUBCUTANEOUS INJECTION | Denosumab(Genetical Recombination) |
| 622007102 | H05AA02 | Forteo Subcuteneous Injection | Teriparatide(Genetical Recombination) |
| 622112301 | H05AA02 | Teribone Injection | Teriparatide Acetate |
| 622293101 | H05AA02 | Teribone Injection | Teriparatide Acetate |
| 630011001 | H05AA02 | Forteo Subcuteneous Injection | Teriparatide(Genetical Recombination) |
| 620001904 | G03XC01 | Evista tablet 60mg | Raloxifene Hydrochloride |
| 622003201 | G03XC02 | Viviant tablet 20mg | Bazedoxifene Acetate |
| 622458301 | G03XC01 | Raloxifene tablet 60ｍg | Raloxifene Hydrochloride |
| 622495301 | G03XC01 | Raloxifene tablet 60ｍg | Raloxifene Hydrochloride |
| 622498201 | G03XC01 | Raloxifene tablet 60ｍg | Raloxifene Hydrochloride |
| 622503101 | G03XC01 | Raloxifene tablet 60ｍg | Raloxifene Hydrochloride |
| 622514501 | G03XC01 | Raloxifene tablet 60ｍg | Raloxifene Hydrochloride |
| 622520601 | G03XC01 | Raloxifene tablet 60ｍg | Raloxifene Hydrochloride |
| 622532201 | G03XC01 | Raloxifene tablet 60ｍg | Raloxifene Hydrochloride |
| 622554801 | G03XC01 | Raloxifene tablet 60ｍg | Raloxifene Hydrochloride |
| 622568701 | G03XC01 | Raloxifene tablet 60ｍg | Raloxifene Hydrochloride |
| 610406085 | A11CC04 | Calcitarol capsule | Calcitriol |
| 610406086 | A11CC04 | Calcitarol capsule 0.5μg | Calcitriol |
| 610406089 | A11CC04 | Caldemine tablet | Calcitriol |
| 610406207 | A11CC04 | TORUSITORIN capsule | Calcitriol |
| 610406208 | A11CC04 | TORUSITORIN capsule 0.5μg | Calcitriol |
| 610406252 | A11CC04 | Hipoteriol capsule | Calcitriol |
| 610406253 | A11CC04 | Hipoteriol capsule 0.5μg | Calcitriol |
| 610461011 | A11CC03 | Alfacalcidol Capsule 0.5μg | Alfacalcidol |
| 610461012 | A11CC03 | Alfacalcidol tablet 0.5μg | Alfacalcidol |
| 610461013 | A11CC03 | Alfacalcidol Capsule | Alfacalcidol |
| 610461014 | A11CC03 | Alfacalcidol tablet | Alfacalcidol |
| 610461109 | A11CC04 | Calcitarol capsule 0.25μg | Calcitriol |
| 610461111 | A11CC04 | Calcitarol capsule 0.5μg | Calcitriol |
| 613110001 | A11CC03 | Alfacalcidol Capsule | Alfacalcidol |
| 613110002 | A11CC03 | Alfacalcidol Capsule | Alfacalcidol |
| 613110003 | A11CC03 | Alfacalcidol Capsule | Alfacalcidol |
| 613110004 | A11CC03 | Alfacalcidol Capsule | Alfacalcidol |
| 613110017 | A11CC04 | Rocaltorol capsule 0.25 µg | Calcitriol |
| 613110018 | A11CC04 | Rocaltorol capsule 0.5 µg | Calcitriol |
| 613110024 | A11CC03 | Alcadol capsule 0.5 µg | Alfacalcidol |
| 613110032 | A11CC03 | Toyofarol capsule 0.25 µg | Alfacalcidol |
| 613110034 | A11CC03 | Warkmin capsule 0.25µg | Alfacalcidol |
| 613110036 | A11CC03 | Alcadol capsule 0.5 µg | Alfacalcidol |
| 613110037 | A11CC03 | Alsiodol capsule 0.5µg | Alfacalcidol |
| 613110039 | A11CC03 | Arowtohl capsule 0.5 µg | Alfacalcidol |
| 613110046 | A11CC03 | Toyofarol capsule 0.5 µg | Alfacalcidol |
| 613110050 | A11CC03 | Warkmin capsule 0.5µg | Alfacalcidol |
| 613110052 | A11CC03 | Alcadol capsule 1.0 µg | Alfacalcidol |
| 613110053 | A11CC03 | Alsiodol capsule 1μg | Alfacalcidol |
| 613110055 | A11CC03 | Arowtohl capsule 1.0 µg | Alfacalcidol |
| 613110063 | A11CC03 | Toyofarol capsule 1.0 µg | Alfacalcidol |
| 613110067 | A11CC03 | Warkmin capsule 1.0 µg | Alfacalcidol |
| 620003508 | A11CC03 | Alfarol powder 1μg/g | Alfacalcidol |
| 620003509 | A11CC03 | Alfarol Liquid 0.5µg/mL | Alfacalcidol |
| 620006882 | A11CC03 | Calcitamin capsule 0.25 µg | Alfacalcidol |
| 620006884 | A11CC03 | Calfina tablet 0.25µg | Alfacalcidol |
| 620008297 | A11CC03 | Calcitamin capsule 0.5 μg | Alfacalcidol |
| 620008298 | A11CC03 | Calcitamin capsule 1.0 μg | Alfacalcidol |
| 620008299 | A11CC03 | Calfina tablet 0.5µg | Alfacalcidol |
| 620008300 | A11CC03 | Calfina tablet 1.0µg | Alfacalcidol |
| 620675301 | A11CC03 | Onealfa 0.25 µg | Alfacalcidol |
| 620675501 | A11CC03 | Onealfa 0.5 µg | Alfacalcidol |
| 620675701 | A11CC03 | Onealfa 1.0 µg | Alfacalcidol |
| 620675904 | A11CC03 | Alfacalcidol capsule 0.25μg | Alfacalcidol |
| 620675906 | A11CC03 | Alfacalcidol capsule 0.25μg | Alfacalcidol |
| 620675910 | A11CC03 | Alfacalcidol capsule 0.25μg | Alfacalcidol |
| 620675913 | A11CC03 | Alfacalcidol capsule 0.25μg | Alfacalcidol |
| 620675922 | A11CC03 | Alfacalcidol capsule 0.25μg | Alfacalcidol |
| 620675928 | A11CC03 | Alfacalcidol capsule 0.25μg | Alfacalcidol |
| 620675929 | A11CC03 | Alfacalcidol capsule 0.25μg | Alfacalcidol |
| 620675931 | A11CC03 | Alfacalcidol capsule 0.25μg | Alfacalcidol |
| 620676305 | A11CC03 | Alfacalcidol capsule 0.25μg | Alfacalcidol |
| 620676307 | A11CC03 | Alfacalcidol capsule 0.5μg | Alfacalcidol |
| 620676313 | A11CC03 | Alfacalcidol capsule 0.5μg | Alfacalcidol |
| 620676317 | A11CC03 | Alfacalcidol capsule 0.5μg | Alfacalcidol |
| 620676327 | A11CC03 | Alfacalcidol capsule 0.5μg | Alfacalcidol |
| 620676337 | A11CC03 | Alfacalcidol capsule 0.5μg | Alfacalcidol |
| 620676345 | A11CC03 | Alfacalcidol capsule 0.5μg | Alfacalcidol |
| 620676346 | A11CC03 | Alfacalcidol capsule 0.5μg | Alfacalcidol |
| 620676348 | A11CC03 | Alfacalcidol capsule 0.5μg | Alfacalcidol |
| 620676806 | A11CC03 | Alfacalcidol capsule 1.0μg | Alfacalcidol |
| 620676808 | A11CC03 | Alfacalcidol capsule 1.0μg | Alfacalcidol |
| 620676815 | A11CC03 | Alfacalcidol capsule 1.0μg | Alfacalcidol |
| 620676819 | A11CC03 | Alfacalcidol capsule 1.0μg | Alfacalcidol |
| 620676829 | A11CC03 | Alfacalcidol capsule 1.0μg | Alfacalcidol |
| 620676837 | A11CC03 | Alfacalcidol capsule 1.0μg | Alfacalcidol |
| 620676845 | A11CC03 | Alfacalcidol capsule 1.0μg | Alfacalcidol |
| 620676846 | A11CC03 | Alfacalcidol capsule 1.0μg | Alfacalcidol |
| 620676848 | A11CC03 | Alfacalcidol capsule 1.0μg | Alfacalcidol |
| 620677501 | A11CC03 | Onealfa Liquid 0.5µg | Alfacalcidol |
| 620678104 | A11CC04 | Calcitriol capsule 0.25µg | Calcitriol |
| 620678130 | A11CC04 | Calcitriol capsule 0.25µg | Calcitriol |
| 620678136 | A11CC04 | Calcitriol capsule 0.25µg | Calcitriol |
| 620678704 | A11CC04 | Calcitriol capsule 0.5µg | Calcitriol |
| 620678729 | A11CC04 | Calcitriol capsule 0.5µg | Calcitriol |
| 620678734 | A11CC04 | Calcitriol capsule 0.5µg | Calcitriol |
| 620678738 | A11CC04 | Calcitriol capsule 0.5µg | Calcitriol |
| 621576003 | A11CC04 | Calcitriol capsule 0.25µg | Calcitriol |
| 621957501 | A11CC03 | Arowtohl capsule 0.25 µg | Alfacalcidol |
| 621960302 | A11CC03 | alfacalcidol capsule 3µg | Alfacalcidol |
| 621976501 | A11CC03 | alfacalcidol capsule 3µg | Alfacalcidol |
| 621976800 | A11CC03 | alfacalcidol capsule 3µg | Alfacalcidol |
| 621976803 | A11CC03 | alfacalcidol capsule 3µg | Alfacalcidol |
| 621979202 | A11CC03 | alfacalcidol capsule 3µg | Alfacalcidol |
| 621983601 | A11CC03 | Toyofarol capsule 3µg | Alfacalcidol |
| 621990302 | A11CC03 | alfacalcidol capsule 3µg | Alfacalcidol |
| 621996701 | A11CC03 | alsiodol capsule 3µg | Alfacalcidol |
| 621996802 | A11CC03 | alfacalcidol capsule 3µg | Alfacalcidol |
| 621996902 | A11CC03 | alfacalcidol capsule 3µg | Alfacalcidol |
| 622001601 | A11CC03 | Calfina capsule 3µg | Alfacalcidol |
| 622004002 | A11CC03 | alfacalcidol capsule 3µg | Alfacalcidol |
| 622037402 | A11CC03 | alfacalcidol capsule 3µg | Alfacalcidol |
| 622037701 | A11CC03 | Alcadol capsule 30 µg | Alfacalcidol |
| 622042502 | ｰ | Edirol capsule 0.5 µg | Eldecalcitol |
| 622042602 | ｰ | Edirol capsule 0.75 µg | Eldecalcitol |
| 622074901 | A11CC03 | alfacalcidol capsule 0.25µg | Alfacalcidol |
| 622082601 | A11CC04 | Caldemine capsule 0.5µg | Calcitriol |
| 622083201 | A11CC03 | Alsiodol capsule 0.25μg | Alfacalcidol |
| 622310300 | A11CC03 | alfacalcidol capsule 0.25µg | Alfacalcidol |
| 622310400 | A11CC03 | alfacalcidol tablet 0.25µg | Alfacalcidol |

**Table S5. Ministry of Health, Labor and Welfare standard code for prescription of drugs; Antidementia drugs**

| **Code** | **ATC codes** | **Drug Name** | **General Name** |
| --- | --- | --- | --- |
| 620005909 | N06D | Aricept® D Tablets 10mg | Donepezil Hydrochloride |
| 620001922 | N06D | Aricept® D Tablets 3mg | Donepezil Hydrochloride |
| 620001923 | N06D | Aricept® D Tablets 5mg | Donepezil Hydrochloride |
| 622256701 | N06D | Aricept® Dry Syrup 1% | Donepezil Hydrochloride |
| 610453016 | N06D | Aricept® Powder 10mg | Donepezil Hydrochloride |
| 620005908 | N06D | Aricept® Tablets 10mg | Donepezil Hydrochloride |
| 610432042 | N06D | Aricept® Tablets 3mg | Donepezil Hydrochloride |
| 610432043 | N06D | Aricept® Tablets 5mg | Donepezil Hydrochloride |
| 621958001 | N06D | Aricept® Oral Jerry 10mg | Donepezil Hydrochloride |
| 621957801 | N06D | Aricept® Oral Jerry 3mg | Donepezil Hydrochloride |
| 621957901 | N06D | Aricept® Oral Jerry 5mg | Donepezil Hydrochloride |
| 622473800 | N06D | Donepezil Hydrochloride 10mg | Donepezil Hydrochloride |
| 622291601 | N06D | Donepezil Hydrochloride OD Film tablet 10mg | Donepezil Hydrochloride |
| 622125501 | N06D | Donepezil Hydrochloride OD Film tablet 3mg | Donepezil Hydrochloride |
| 622125601 | N06D | Donepezil Hydrochloride OD File tablet 5mg | Donepezil Hydrochloride |
| 622287001 | N06D | Donepezil Hydrochloride OD Tablet 10mg | Donepezil Hydrochloride |
| 622289901 | N06D | Donepezil Hydrochloride OD Tablet 10mg | Donepezil Hydrochloride |
| 622297901 | N06D | Donepezil Hydrochloride OD Tablet 10mg | Donepezil Hydrochloride |
| 622288701 | N06D | Donepezil Hydrochloride OD Tablet 10mg | Donepezil Hydrochloride |
| 622304101 | N06D | Donepezil Hydrochloride OD Tablet 10mg | Donepezil Hydrochloride |
| 622294001 | N06D | Donepezil Hydrochloride OD Tablet 10mg | Donepezil Hydrochloride |
| 622277301 | N06D | Donepezil Hydrochloride OD Tablet 10mg | Donepezil Hydrochloride |
| 622303101 | N06D | Donepezil Hydrochloride OD Tablet 10mg | Donepezil Hydrochloride |
| 622284501 | N06D | Donepezil Hydrochloride OD Tablet 10mg | Donepezil Hydrochloride |
| 622269801 | N06D | Donepezil Hydrochloride OD Tablet 10mg | Donepezil Hydrochloride |
| 622268301 | N06D | Donepezil Hydrochloride OD Tablet 10mg | Donepezil Hydrochloride |
| 622275901 | N06D | Donepezil Hydrochloride OD Tablet 10mg | Donepezil Hydrochloride |
| 622277701 | N06D | Donepezil Hydrochloride OD Tablet 10mg | Donepezil Hydrochloride |
| 622595801 | N06D | Donepezil Hydrochloride OD Tablet 10mg | Donepezil Hydrochloride |
| 622287201 | N06D | Donepezil Hydrochloride OD Tablet 10mg | Donepezil Hydrochloride |
| 622295701 | N06D | Donepezil Hydrochloride OD Tablet 10mg | Donepezil Hydrochloride |
| 622289701 | N06D | Donepezil Hydrochloride OD Tablet 10mg | Donepezil Hydrochloride |
| 622274801 | N06D | Donepezil Hydrochloride OD Tablet 10mg | Donepezil Hydrochloride |
| 622273801 | N06D | Donepezil Hydrochloride OD Tablet 10mg | Donepezil Hydrochloride |
| 622284301 | N06D | Donepezil Hydrochloride OD Tablet 10mg | Donepezil Hydrochloride |
| 622280001 | N06D | Donepezil Hydrochloride OD Tablet 10mg | Donepezil Hydrochloride |
| 622284901 | N06D | Donepezil Hydrochloride OD Tablet 10mg | Donepezil Hydrochloride |
| 622269001 | N06D | Donepezil Hydrochloride OD Tablet 10mg | Donepezil Hydrochloride |
| 622298901 | N06D | Donepezil Hydrochloride OD Tablet 10mg | Donepezil Hydrochloride |
| 622270301 | N06D | Donepezil Hydrochloride OD Tablet 10mg | Donepezil Hydrochloride |
| 622282501 | N06D | Donepezil Hydrochloride OD Tablet 10mg | Donepezil Hydrochloride |
| 622286001 | N06D | Donepezil Hydrochloride OD Tablet 10mg | Donepezil Hydrochloride |
| 622170501 | N06D | Donepezil Hydrochloride OD Tablet 3mg | Donepezil Hydrochloride |
| 622086901 | N06D | Donepezil Hydrochloride OD Tablet 3mg | Donepezil Hydrochloride |
| 622115101 | N06D | Donepezil Hydrochloride OD Tablet 3mg | Donepezil Hydrochloride |
| 622113801 | N06D | Donepezil Hydrochloride OD Tablet 3mg | Donepezil Hydrochloride |
| 622128601 | N06D | Donepezil Hydrochloride OD Tablet 3mg | Donepezil Hydrochloride |
| 622124201 | N06D | Donepezil Hydrochloride OD Tablet 3mg | Donepezil Hydrochloride |
| 622155501 | N06D | Donepezil Hydrochloride OD Tablet 3mg | Donepezil Hydrochloride |
| 622105501 | N06D | Donepezil Hydrochloride OD Tablet 3mg | Donepezil Hydrochloride |
| 622097502 | N06D | Donepezil Hydrochloride OD Tablet 3mg | Donepezil Hydrochloride |
| 622116101 | N06D | Donepezil Hydrochloride OD Tablet 3mg | Donepezil Hydrochloride |
| 622113301 | N06D | Donepezil Hydrochloride OD Tablet 3mg | Donepezil Hydrochloride |
| 622091901 | N06D | Donepezil Hydrochloride OD Tablet 3mg | Donepezil Hydrochloride |
| 622138701 | N06D | Donepezil Hydrochloride OD Tablet 3mg | Donepezil Hydrochloride |
| 622595601 | N06D | Donepezil Hydrochloride OD Tablet 3mg | Donepezil Hydrochloride |
| 622117801 | N06D | Donepezil Hydrochloride OD Tablet 3mg | Donepezil Hydrochloride |
| 622099601 | N06D | Donepezil Hydrochloride OD Tablet 3mg | Donepezil Hydrochloride |
| 622214801 | N06D | Donepezil Hydrochloride OD Tablet 3mg | Donepezil Hydrochloride |
| 622094201 | N06D | Donepezil Hydrochloride OD Tablet 3mg | Donepezil Hydrochloride |
| 622095001 | N06D | Donepezil Hydrochloride OD Tablet 3mg | Donepezil Hydrochloride |
| 622101202 | N06D | Donepezil Hydrochloride OD Tablet 3mg | Donepezil Hydrochloride |
| 622109901 | N06D | Donepezil Hydrochloride OD Tablet 3mg | Donepezil Hydrochloride |
| 622120001 | N06D | Donepezil Hydrochloride OD Tablet 3mg | Donepezil Hydrochloride |
| 622120501 | N06D | Donepezil Hydrochloride OD Tablet 3mg | Donepezil Hydrochloride |
| 622123201 | N06D | Donepezil Hydrochloride OD Tablet 3mg | Donepezil Hydrochloride |
| 622128701 | N06D | Donepezil Hydrochloride OD Tablet 3mg | Donepezil Hydrochloride |
| 622107401 | N06D | Donepezil Hydrochloride OD Tablet 3mg | Donepezil Hydrochloride |
| 622112701 | N06D | Donepezil Hydrochloride OD Tablet 3mg | Donepezil Hydrochloride |
| 622170601 | N06D | Donepezil Hydrochloride OD Tablet 5mg | Donepezil Hydrochloride |
| 622087001 | N06D | Donepezil Hydrochloride OD Tablet 5mg | Donepezil Hydrochloride |
| 622115201 | N06D | Donepezil Hydrochloride OD Tablet 5mg | Donepezil Hydrochloride |
| 622113901 | N06D | Donepezil Hydrochloride OD Tablet 5mg | Donepezil Hydrochloride |
| 622128801 | N06D | Donepezil Hydrochloride OD Tablet 5mg | Donepezil Hydrochloride |
| 622124301 | N06D | Donepezil Hydrochloride OD Tablet 5mg | Donepezil Hydrochloride |
| 622155601 | N06D | Donepezil Hydrochloride OD Tablet 5mg | Donepezil Hydrochloride |
| 622105601 | N06D | Donepezil Hydrochloride OD Tablet 5mg | Donepezil Hydrochloride |
| 622097602 | N06D | Donepezil Hydrochloride OD Tablet 5mg | Donepezil Hydrochloride |
| 622116201 | N06D | Donepezil Hydrochloride OD Tablet 5mg | Donepezil Hydrochloride |
| 622113401 | N06D | Donepezil Hydrochloride OD Tablet 5mg | Donepezil Hydrochloride |
| 622092001 | N06D | Donepezil Hydrochloride OD Tablet 5mg | Donepezil Hydrochloride |
| 622138801 | N06D | Donepezil Hydrochloride OD Tablet 5mg | Donepezil Hydrochloride |
| 622595701 | N06D | Donepezil Hydrochloride OD Tablet 5mg | Donepezil Hydrochloride |
| 622117901 | N06D | Donepezil Hydrochloride OD Tablet 5mg | Donepezil Hydrochloride |
| 622099701 | N06D | Donepezil Hydrochloride OD Tablet 5mg | Donepezil Hydrochloride |
| 622214901 | N06D | Donepezil Hydrochloride OD Tablet 5mg | Donepezil Hydrochloride |
| 622094301 | N06D | Donepezil Hydrochloride OD Tablet 5mg | Donepezil Hydrochloride |
| 622095101 | N06D | Donepezil Hydrochloride OD Tablet 5mg | Donepezil Hydrochloride |
| 622101302 | N06D | Donepezil Hydrochloride OD Tablet 5mg | Donepezil Hydrochloride |
| 622110001 | N06D | Donepezil Hydrochloride OD Tablet 5mg | Donepezil Hydrochloride |
| 622120101 | N06D | Donepezil Hydrochloride OD Tablet 5mg | Donepezil Hydrochloride |
| 622120601 | N06D | Donepezil Hydrochloride OD Tablet 5mg | Donepezil Hydrochloride |
| 622123301 | N06D | Donepezil Hydrochloride OD Tablet 5mg | Donepezil Hydrochloride |
| 622128901 | N06D | Donepezil Hydrochloride OD Tablet 5mg | Donepezil Hydrochloride |
| 622107501 | N06D | Donepezil Hydrochloride OD Tablet 5mg | Donepezil Hydrochloride |
| 622112801 | N06D | Donepezil Hydrochloride OD Tablet 5mg | Donepezil Hydrochloride |
| 622092301 | N06D | Donepezil Hydrochloride Powder 0.5% | Donepezil Hydrochloride |
| 622099801 | N06D | Donepezil Hydrochloride Powder 0.5% | Donepezil Hydrochloride |
| 622129001 | N06D | Donepezil Hydrochloride Powder 0.5% | Donepezil Hydrochloride |
| 622287901 | N06D | Donepezil Hydrochloride Tablet 10mg | Donepezil Hydrochloride |
| 622286901 | N06D | Donepezil Hydrochloride Tablet 10mg | Donepezil Hydrochloride |
| 622290001 | N06D | Donepezil Hydrochloride Tablet 10mg | Donepezil Hydrochloride |
| 622297801 | N06D | Donepezil Hydrochloride Tablet 10mg | Donepezil Hydrochloride |
| 622288801 | N06D | Donepezil Hydrochloride Tablet 10mg | Donepezil Hydrochloride |
| 622294101 | N06D | Donepezil Hydrochloride Tablet 10mg | Donepezil Hydrochloride |
| 622277201 | N06D | Donepezil Hydrochloride Tablet 10mg | Donepezil Hydrochloride |
| 622303001 | N06D | Donepezil Hydrochloride Tablet 10mg | Donepezil Hydrochloride |
| 622304201 | N06D | Donepezil Hydrochloride Tablet 10mg | Donepezil Hydrochloride |
| 622284401 | N06D | Donepezil Hydrochloride Tablet 10mg | Donepezil Hydrochloride |
| 622273401 | N06D | Donepezil Hydrochloride Tablet 10mg | Donepezil Hydrochloride |
| 622276001 | N06D | Donepezil Hydrochloride Tablet 10mg | Donepezil Hydrochloride |
| 622277601 | N06D | Donepezil Hydrochloride Tablet 10mg | Donepezil Hydrochloride |
| 622566201 | N06D | Donepezil Hydrochloride Tablet 10mg | Donepezil Hydrochloride |
| 622297501 | N06D | Donepezil Hydrochloride Tablet 10mg | Donepezil Hydrochloride |
| 622295801 | N06D | Donepezil Hydrochloride Tablet 10mg | Donepezil Hydrochloride |
| 622274701 | N06D | Donepezil Hydrochloride Tablet 10mg | Donepezil Hydrochloride |
| 622273701 | N06D | Donepezil Hydrochloride Tablet 10mg | Donepezil Hydrochloride |
| 622284201 | N06D | Donepezil Hydrochloride Tablet 10mg | Donepezil Hydrochloride |
| 622279901 | N06D | Donepezil Hydrochloride Tablet 10mg | Donepezil Hydrochloride |
| 622268901 | N06D | Donepezil Hydrochloride Tablet 10mg | Donepezil Hydrochloride |
| 622298601 | N06D | Donepezil Hydrochloride Tablet 10mg | Donepezil Hydrochloride |
| 622270201 | N06D | Donepezil Hydrochloride Tablet 10mg | Donepezil Hydrochloride |
| 622282401 | N06D | Donepezil Hydrochloride Tablet 10mg | Donepezil Hydrochloride |
| 622285901 | N06D | Donepezil Hydrochloride Tablet 10mg | Donepezil Hydrochloride |
| 622081201 | N06D | Donepezil Hydrochloride Tablet 3mg | Donepezil Hydrochloride |
| 622115901 | N06D | Donepezil Hydrochloride Tablet 3mg | Donepezil Hydrochloride |
| 622086701 | N06D | Donepezil Hydrochloride Tablet 3mg | Donepezil Hydrochloride |
| 622114901 | N06D | Donepezil Hydrochloride Tablet 3mg | Donepezil Hydrochloride |
| 622113601 | N06D | Donepezil Hydrochloride Tablet 3mg | Donepezil Hydrochloride |
| 622124001 | N06D | Donepezil Hydrochloride Tablet 3mg | Donepezil Hydrochloride |
| 622155301 | N06D | Donepezil Hydrochloride Tablet 3mg | Donepezil Hydrochloride |
| 622105301 | N06D | Donepezil Hydrochloride Tablet 3mg | Donepezil Hydrochloride |
| 622126401 | N06D | Donepezil Hydrochloride Tablet 3mg | Donepezil Hydrochloride |
| 622097302 | N06D | Donepezil Hydrochloride Tablet 3mg | Donepezil Hydrochloride |
| 622116301 | N06D | Donepezil Hydrochloride Tablet 3mg | Donepezil Hydrochloride |
| 622092101 | N06D | Donepezil Hydrochloride Tablet 3mg | Donepezil Hydrochloride |
| 622087201 | N06D | Donepezil Hydrochloride Tablet 3mg | Donepezil Hydrochloride |
| 622565901 | N06D | Donepezil Hydrochloride Tablet 3mg | Donepezil Hydrochloride |
| 622117601 | N06D | Donepezil Hydrochloride Tablet 3mg | Donepezil Hydrochloride |
| 622099901 | N06D | Donepezil Hydrochloride Tablet 3mg | Donepezil Hydrochloride |
| 622126701 | N06D | Donepezil Hydrochloride Tablet 3mg | Donepezil Hydrochloride |
| 622094001 | N06D | Donepezil Hydrochloride Tablet 3mg | Donepezil Hydrochloride |
| 622094801 | N06D | Donepezil Hydrochloride Tablet 3mg | Donepezil Hydrochloride |
| 622101002 | N06D | Donepezil Hydrochloride Tablet 3mg | Donepezil Hydrochloride |
| 622109701 | N06D | Donepezil Hydrochloride Tablet 3mg | Donepezil Hydrochloride |
| 622120301 | N06D | Donepezil Hydrochloride Tablet 3mg | Donepezil Hydrochloride |
| 622123001 | N06D | Donepezil Hydrochloride Tablet 3mg | Donepezil Hydrochloride |
| 622129101 | N06D | Donepezil Hydrochloride Tablet 3mg | Donepezil Hydrochloride |
| 622107201 | N06D | Donepezil Hydrochloride Tablet 3mg | Donepezil Hydrochloride |
| 622112501 | N06D | Donepezil Hydrochloride Tablet 3mg | Donepezil Hydrochloride |
| 622081301 | N06D | Donepezil Hydrochloride Tablet 5mg | Donepezil Hydrochloride |
| 622116001 | N06D | Donepezil Hydrochloride Tablet 5mg | Donepezil Hydrochloride |
| 622086801 | N06D | Donepezil Hydrochloride Tablet 5mg | Donepezil Hydrochloride |
| 622115001 | N06D | Donepezil Hydrochloride Tablet 5mg | Donepezil Hydrochloride |
| 622113701 | N06D | Donepezil Hydrochloride Tablet 5mg | Donepezil Hydrochloride |
| 622124101 | N06D | Donepezil Hydrochloride Tablet 5mg | Donepezil Hydrochloride |
| 622155401 | N06D | Donepezil Hydrochloride Tablet 5mg | Donepezil Hydrochloride |
| 622105401 | N06D | Donepezil Hydrochloride Tablet 5mg | Donepezil Hydrochloride |
| 622126501 | N06D | Donepezil Hydrochloride Tablet 5mg | Donepezil Hydrochloride |
| 622097402 | N06D | Donepezil Hydrochloride Tablet 5mg | Donepezil Hydrochloride |
| 622116401 | N06D | Donepezil Hydrochloride Tablet 5mg | Donepezil Hydrochloride |
| 622092201 | N06D | Donepezil Hydrochloride Tablet 5mg | Donepezil Hydrochloride |
| 622087301 | N06D | Donepezil Hydrochloride Tablet 5mg | Donepezil Hydrochloride |
| 622566101 | N06D | Donepezil Hydrochloride Tablet 5mg | Donepezil Hydrochloride |
| 622117701 | N06D | Donepezil Hydrochloride Tablet 5mg | Donepezil Hydrochloride |
| 622100001 | N06D | Donepezil Hydrochloride Tablet 5mg | Donepezil Hydrochloride |
| 622126801 | N06D | Donepezil Hydrochloride Tablet 5mg | Donepezil Hydrochloride |
| 622094101 | N06D | Donepezil Hydrochloride Tablet 5mg | Donepezil Hydrochloride |
| 622094901 | N06D | Donepezil Hydrochloride Tablet 5mg | Donepezil Hydrochloride |
| 622101102 | N06D | Donepezil Hydrochloride Tablet 5mg | Donepezil Hydrochloride |
| 622109801 | N06D | Donepezil Hydrochloride Tablet 5mg | Donepezil Hydrochloride |
| 622120401 | N06D | Donepezil Hydrochloride Tablet 5mg | Donepezil Hydrochloride |
| 622123101 | N06D | Donepezil Hydrochloride Tablet 5mg | Donepezil Hydrochloride |
| 622129201 | N06D | Donepezil Hydrochloride Tablet 5mg | Donepezil Hydrochloride |
| 622107301 | N06D | Donepezil Hydrochloride Tablet 5mg | Donepezil Hydrochloride |
| 622112601 | N06D | Donepezil Hydrochloride Tablet 5mg | Donepezil Hydrochloride |
| 622294201 | N06D | Donepezil Hydrochloride Oral Jerry 10mg | Donepezil Hydrochloride |
| 622270401 | N06D | Donepezil Hydrochloride Oral Jerry 10mg | Donepezil Hydrochloride |
| 622165101 | N06D | Donepezil Hydrochloride Oral Jerry 3mg | Donepezil Hydrochloride |
| 622173201 | N06D | Donepezil Hydrochloride Oral Jerry 3mg | Donepezil Hydrochloride |
| 622165201 | N06D | Donepezil Hydrochloride Oral Jerry 5mg | Donepezil Hydrochloride |
| 622173301 | N06D | Donepezil Hydrochloride Oral Jerry 5mg | Donepezil Hydrochloride |
| 622274101 | N06D | Donepezil Hydrochloride Liquid 10mg | Donepezil Hydrochloride |
| 622279801 | N06D | Donepezil Hydrochloride Liquid 10mg | Donepezil Hydrochloride |
| 622273901 | N06D | Donepezil Hydrochloride Liquid 3mg | Donepezil Hydrochloride |
| 622142101 | N06D | Donepezil Hydrochloride Liquid 3mg | Donepezil Hydrochloride |
| 622274001 | N06D | Donepezil Hydrochloride Liquid 5mg | Donepezil Hydrochloride |
| 622142201 | N06D | Donepezil Hydrochloride Liquid 5mg | Donepezil Hydrochloride |
| 622306101 | N06DX01 | Memary® oral OD tablets 10mg | Memantine Hydrochloride |
| 622306201 | N06DX01 | Memary® oral OD tablets 20mg | Memantine Hydrochloride |
| 622306001 | N06DX01 | Memary® oral OD tablets 5mg | Memantine Hydrochloride |
| 622047201 | N06DX01 | Memary® tablets 10mg | Memantine Hydrochloride |
| 622047301 | N06DX01 | Memary® tablets 20mg | Memantine Hydrochloride |
| 622047101 | N06DX01 | Memary® tablets 5mg | Memantine Hydrochloride |
| 622085001 | N06DA03 | RIVASTACH Ⓡ Patches 13.5mg | Rivastigmine |
| 622085101 | N06DA03 | RIVASTACH Ⓡ Patches 18mg | Rivastigmine |
| 622084801 | N06DA03 | RIVASTACH Ⓡ Patches 4.5mg | Rivastigmine |
| 622084901 | N06DA03 | RIVASTACH Ⓡ Patches 9mg | Rivastigmine |
| 622046301 | N06DA04 | REMINYLⓇ OD Tablets 12mg | Galantamine Hydrobromide |
| 622046101 | N06DA04 | REMINYLⓇ OD Tablets 4mg | Galantamine Hydrobromide |
| 622046201 | N06DA04 | REMINYLⓇ OD Tablets 8mg | Galantamine Hydrobromide |
| 622046001 | N06DA04 | REMINYLⓇ Tablets 12mg | Galantamine Hydrobromide |
| 622045801 | N06DA04 | REMINYLⓇ Tablets 4mg | Galantamine Hydrobromide |
| 622045901 | N06DA04 | REMINYLⓇ Tablets 8mg | Galantamine Hydrobromide |
| 622046401 | N06DA04 | REMINYLⓇ Oral Solution 4mg/mL | Galantamine Hydrobromide |

**Figure S1. The numbers of individuals who received each antidementia drug among 496 DLB patients who received memantine or ChEIs, except donepezil, at least one time**

**
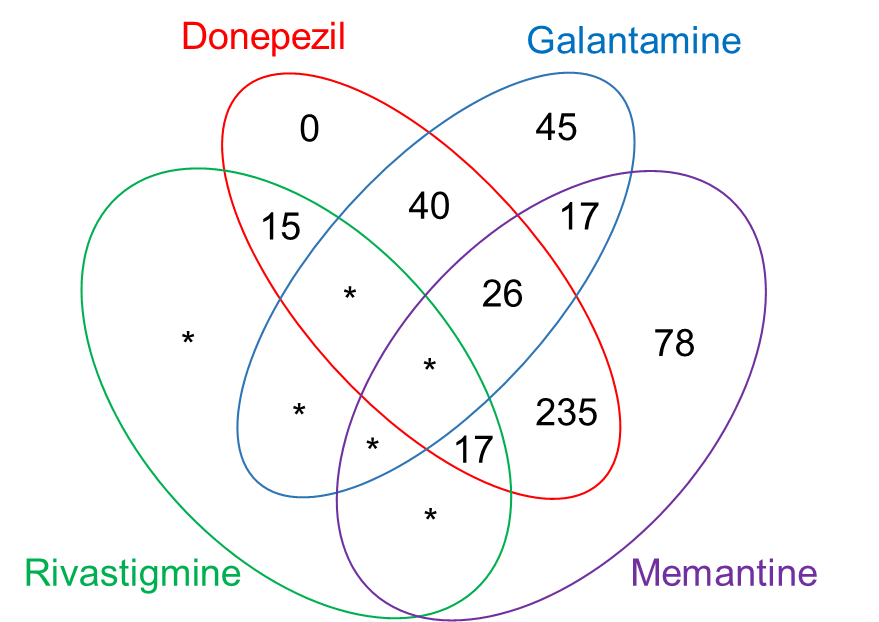
**

The Venn diagram displays the numbers of individuals who received each antidementia drug at least one time during 3 years among 496 patients with DLB, who received some antidementia drug except donepezil. (Refer to Figure 1, neither donepezil group nor drug-naïve group.) Asterisk marks in the figure mean <10, and the exact numbers are not shown because results in which the number of any unit is <10 are not allowed to be published.

Abbreviations: *DLB dementia with Lewy bodies*
